# Large Rhinophyma Treated by Surgical Excision and Electrocautery

**DOI:** 10.1155/2019/2395619

**Published:** 2019-07-25

**Authors:** Nabeel K. Al Hamzawi, Salih M. Al Baaj

**Affiliations:** ^1^Department of Dermatology, Diwaniyah Teaching Hospital, Diwaniyah, Iraq; ^2^Department of Plastic Surgery, Diwaniyah Teaching Hospital, Diwaniyah, Iraq

## Abstract

Rhinophyma is a benign condition characterized by a large, bulbous nose with prominent pores. It is commonly associated with untreated cases of rosacea. The disease can carry a substantial psychological impact that causes patients to seek advice about how to improve their physical appearance. Many treatment options are available for rhinophyma, but there is no standard treatment protocol. Here, we describe the case of a 65-year-old man with a large rhinophyma that caused him cosmetic and psychosocial embarrassment. The condition was treated by surgical excision and bipolar electrocautery. No complications occurred after the procedures, and healing was completed 2 weeks later by secondary intention and reepithelialization. A simple surgical removal using a scalpel to shave off the abnormal tissue with electrocauterization of the bleeding points can be considered as a good treatment option for rhinophyma, as it results in an excellent cosmetic outcome and has short recovery time.

## 1. Introduction

Rhinophyma is a benign skin lesion characterized by a large, bulbous, and erythematous-appearing nose. It is considered to be an advanced stage of phymatous rosacea [1]. However, some people have rhinophyma without having rosacea. The diagnosis of rhinophyma is usually based on the clinical appearance of the nose and the history of rosacea. Several treatment options are available such as surgery, dermabrasion, and laser therapy. Here, we describe the case of a large rhinophyma treated successfully by surgical excision and bipolar electrocautery with an excellent cosmetic result.

## 2. Case Report

**Figure 1:**
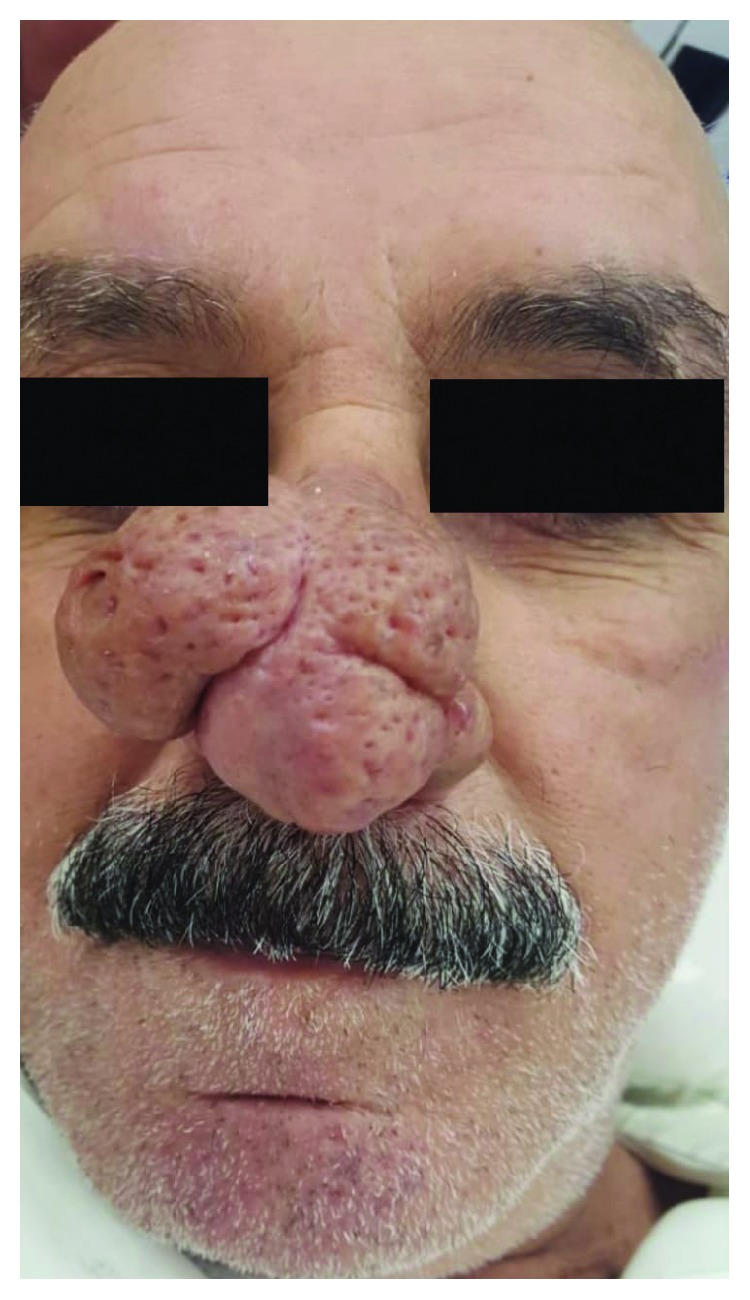
A 65-year-old man with the mid and apical areas of the nose covered with a large lobulated rhinophyma.

**Figure 2:**
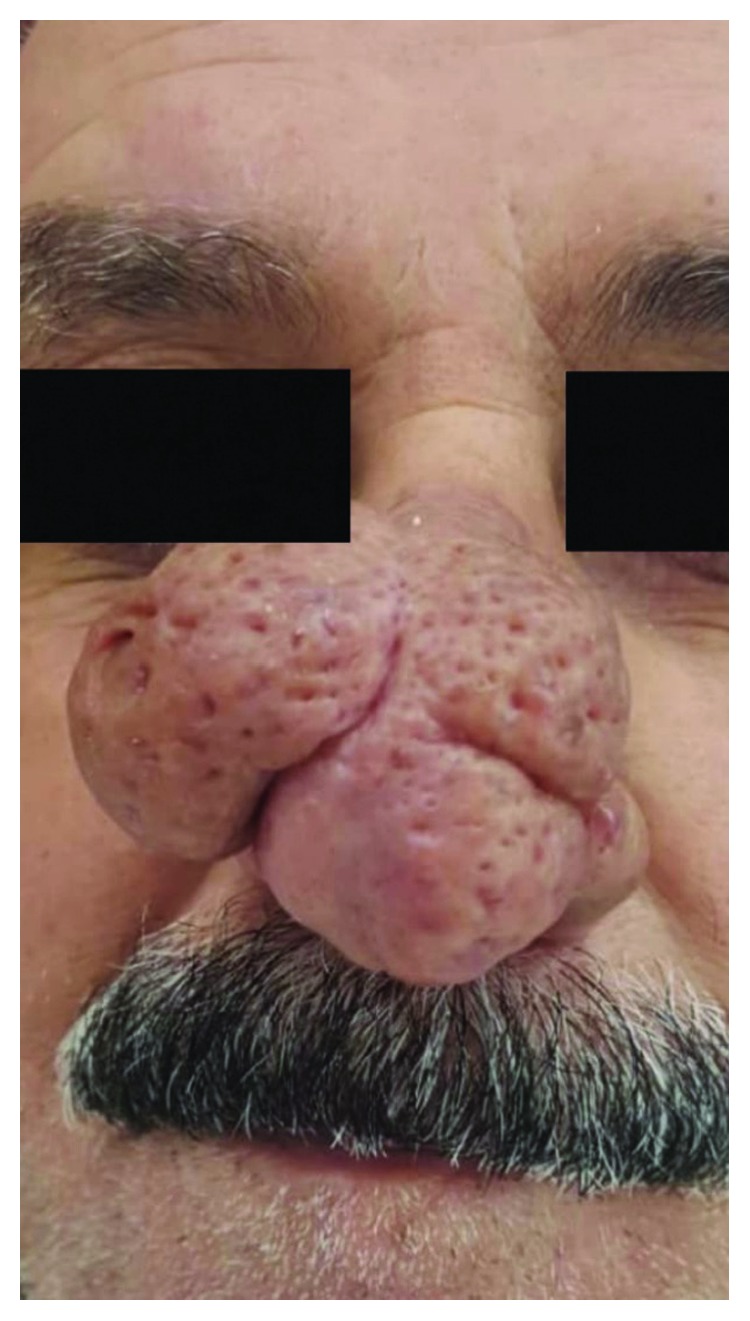
Rhinophyma manifested by three lobules two at the side and one at the apex of the nose with prominent pores and telangiectasia.

A 65-year-old man presented to the outpatient dermatology clinic with a large bulbous nose for the past 5 years. The physical examination showed three hypertrophied lobules covering the tip of the nose with deep pores and fine telangiectasia (Figures 1 and 2). The patient had been complaining of rosacea for years without receiving treatment.

Surgical excision was performed under local anesthesia using 2% lidocaine, which was injected into the nose. After the local anesthetic was infiltrated, the hypertrophied tissue was debulked using a number 10 scalpel. The excess tissue was removed layer by layer, with attention to preserve part of the basal appendages overlying the perichondrium to avoid the possibility of scarring. Hemostasis was achieved using bipolar electrocautery to reduce bleeding from the denuded area at a setting of 10-12 watts. Topical mupirocin ointment was applied to the wound, and the area was secured with a tie-over dressing.

**Figure 3:**
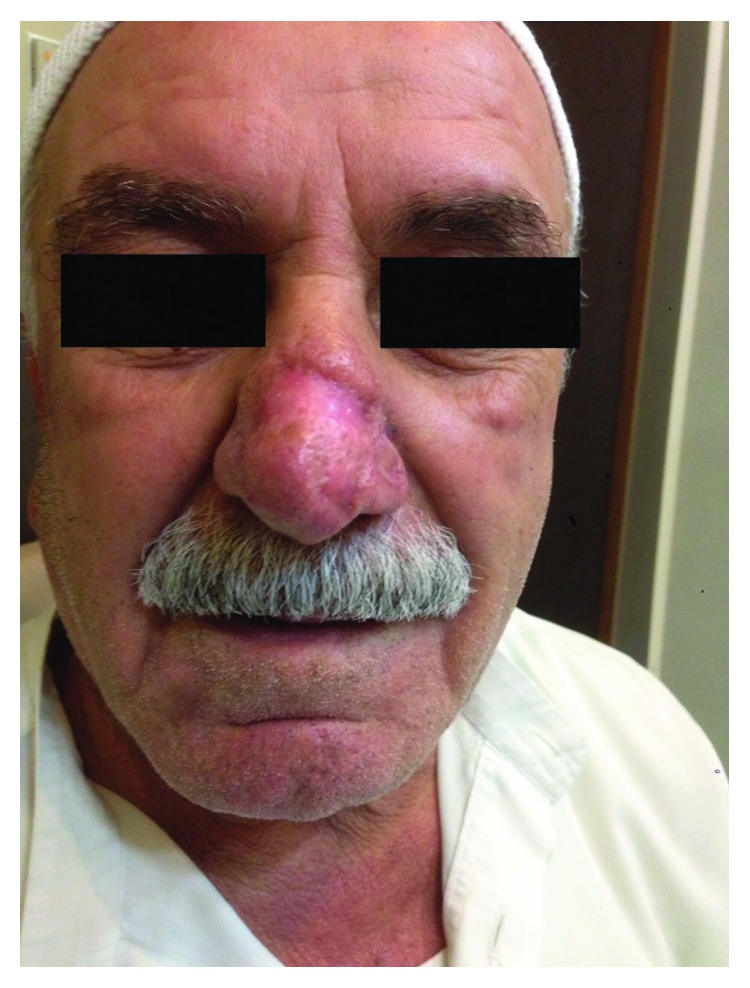
Complete healing by reepithelialization one month after the surgical procedure.

The dressing was removed after 72 hours, and the wound was left exposed to heal by secondary intention. The excised tissue was sent for histopathological study, and the diagnosis of rhinophyma was confirmed. Follow-up was continued with weekly visits until healing was completed by the fourth week (Figure 3).

## 3. Discussion

Rhinophyma is a slow-growing and disfiguring enlargement of the nose that primarily occurs in men aged 50-70 years. Established rhinophyma is characterized by a large bulbous nose, wide pores, thick skin, and telangiectasia [2]. It is considered to be a characteristic of an advanced stage of phymatous rosacea. Phymatous rosacea can also affect the forehead (metophyma), chin (gnathophyma), ears (otophyma), and eyelids (blepharophyma) [3]. The exact cause of rhinophyma is not understood. It is thought to be multifactorial in origin with a primary etiology of unregulated superficial vasodilatation [4, 5]. Several hypotheses include potential roles for environmental factors and microorganisms such as Demodex folliculorum and Helicobacter pylori [6]. There is a misguided belief that it is related to alcohol consumption, with a nickname of the condition “whiskey nose.” There is no link between rhinophyma and alcohol [7]. Rhinophyma can be classified into three clinical varieties: glandular (nasal enlargement due to hyperplasia of the sebaceous glands), fibrous (increased density of the nasal connective tissue with variable sebaceous hyperplasia), and fibroangiomatous (nasal enlargement due to edematous connective tissue with enlarged veins) [8]. A histopathological evaluation can help to exclude disorders that mimic rhinophyma, such as squamous cell carcinoma and angiomatous tumors. Histopathological features of rhinophyma include sebaceous hyperplasia, connective tissue hyperplasia, and signs of chronic inflammation [8].

Treatment of rhinophyma can be very challenging and involve a combination of different treatment options. Medical therapies include oral isotretinoin and antibiotics, such as tetracycline, erythromycin, and topical metronidazole, which may have a positive effect in the early stages of the disease. Oral medications are not usually effective in established rhinophyma; therefore, surgery is often necessary. Surgical treatments are aimed at removing the excess tissue and restoring the natural appearance of the nose; it is considered the standard gold treatment. Surgery, dermabrasion, and debulking and sculpting the tissue using a sinus microdebrider, ablative carbon dioxide laser therapy, electrosurgery, and electrocautery were all considered as treatment options [9, 10]. However, there is no standard protocol to manage rhinophyma. In our case, we used a combined technique of simple surgical excision and bipolar electrocautery as an alternative option to treat large rhinophyma. This procedure is less aggressive, is easy to perform, and leads to a significant result. Furthermore, no complications such as scarring or hyperpigmentation were recorded following this operation after long-term follow-up. In some degree, a similar technique has been reported in the literature. Silva et al. have described two patients with rhinophyma that underwent tangential excision associated with electrocoagulation with good long-term results [11]. The extent of tissue growth and the severity of the condition can help determine the preferred treatment method. The modality of treatment selected and expert skill result in a better outcome.

## 4. Conclusion

Because there is no cure with procedures that are aimed only at removing the overgrowth of rhinophyma and reshaping the nose, surgeons should select the option with the least complications. A simple surgical excision using a scalpel to shave off the abnormal tissue with electrocauterization of the bleeding points can be considered as a good treatment option for rhinophyma, as it results in an excellent cosmetic outcome and has short recovery time.
